# Higher Prevalence of Tooth Loss in People With Abdominal Obesity but Normal Weight: Findings From the United States and Scottish Populations

**DOI:** 10.1002/cre2.70047

**Published:** 2024-11-19

**Authors:** Jing Kang, Harriet Larvin, Sue Pavitt, Jianhua Wu

**Affiliations:** ^1^ Faculty of Dentistry, Oral and Craniofacial Sciences, King's College London London UK; ^2^ Centre of Primary Care, Wolfson Institute of Population Health Queen Mary University of London London UK; ^3^ Dental Translational Clinical Research Unit, School of Dentistry, Faculty of Medicine and Health University of Leeds Leeds West Yorkshire UK

**Keywords:** body mass index, gender difference, obesity, periodontal disease, tooth loss

## Abstract

**Objectives:**

Previous research has shown that people with obesity are at a higher risk of tooth loss; however, it is unclear whether abdominal obesity (e.g., high waist circumference) is associated with tooth loss among individuals without obesity. This study aims to investigate the association between abdominal obesity and tooth loss among people who are not obese.

**Material and Methods:**

Two cross‐sectional surveys were used: the United States' National Health and Nutrition Examination Survey (NHANES) 1999–2012 (*n* = 19,436) and the Scottish Health Survey (SHeS) 2008–2014 (*n* = 4243). Tooth loss was measured by the number of remaining teeth: 20 and over, 1–19, and edentulous. Abdominal obesity was defined by categorizing waist circumference into three levels: normal, high, and very high. Ordinal logistic regression was used to model the association between tooth loss and abdominal obesity.

**Results:**

For people living without obesity, abdominal obesity is associated with a higher prevalence of tooth loss, and the effect is different between women and men. For women, abdominal obesity increased the chance of tooth loss by 64% (odds ratio [OR]: 1.64, 95% confidence interval [CI]: 1.16–2.34) in the NHANES and 196% (OR: 2.96, 95% CI: 1.47–5.97) in the SHeS. For men, abdominal obesity increased the chance of tooth loss by 41% (OR: 1.41, 95% CI: 1.06–1.87) in the NHANES and 65% (OR: 1.65, 95% CI: 1.02–2.73) in the SHeS.

**Conclusions:**

This study indicated that the prevalence of tooth loss is substantially higher in people with abdominal obesity, and this association is distinctively different between men and women. These findings suggest that those who are not obese but have abdominal obesity may be an important target population for oral health prevention strategies.

## 1 Introduction

Obesity is an important global epidemiological issue, and the World Health Organization (WHO) predicted a rise in global adult obesity prevalence to reach 18% in men and exceed 21% in women by 2025 (Collaboration NCDRF, 2016). Although body mass index (BMI) is a rough guide to the degree of adiposity, most studies considered BMI greater than 30 kg/m^2^ as an indicator of obesity, not the fat distribution in the body. Existing evidence has shown that obesity has been linked to systemic inflammatory diseases such as type II diabetes, hypertension, and cardiovascular disease (Falagas and Kompoti 2006; Larvin, Kang, et al. 2021, Larvin et al. 2022a, 2022b). Furthermore, some studies found that truncal fat, as estimated by waist circumference (WC), waist‐to‐hip ratio (WHR), or waist‐to‐height ratio, is positively correlated with metabolic abnormalities, and is associated with a higher risk of mortality (Sahakyan et al. 2015). It is important to understand the potentially harmful effects of abdominal obesity, in addition to high BMI.

Previous studies showed that general obesity, defined by BMI, was associated with a higher risk of oral disease, including periodontal disease and tooth loss (Suvan et al. 2015; Nascimento et al. 2016; Kang et al. 2019; Larvin, Wilmott, et al. 2021). One possible biological mechanism for such association is C‐reactive protein (CRP), a nonspecific inflammatory biomarker linked to many conditions, including infection, periodontitis, cardiovascular disease, diabetes, and obesity. Elevated CRP levels in obesity may contribute to the development of periodontitis, which, along with other inflammatory and environmental factors, can lead to tooth loss or, in severe cases, complete loss of dentition (Linden et al. 2008; Chaffee and Weston 2010; Meisel et al. 2014). The relationship between CRP, obesity, and periodontitis/tooth loss is hypothesized, but further research is needed to confirm the association (Chaffee and Weston 2010; Meisel et al. 2021; Jepsen, Suvan, and Deschner 2020).

It would also be interesting to understand the association between oral disease and abdominal obesity, measured by WC or WHR. These measures provide additional information beyond BMI. Most studies focused on populations with obesity, but individuals with normal BMI and presenting abdominal obesity are understudied.

This study aims to investigate the association between tooth loss and abdominal obesity among individuals without obesity (BMI < 30 kg/m^2^). Two national health surveys from the United States and Scotland were used to demonstrate the similarities and differences in these associations across different countries.

## 2 Methods

### 2.1 Study Design and Participants

This study adheres to the STROBE guidelines for reporting cross‐sectional studies (Uhlig, Menon, and Schmid 2007). This study utilized two cross‐sectional national surveys: the National Health and Nutrition Examination Survey (NHANES) from the United States and the Scottish Health Survey (SHeS) from the United Kingdom (Brown et al. 2015). Both surveys provide reliable dental and health measures, including CRP levels, with large sample sizes. Both surveys are free and publicly accessible via https://www.cdc.gov/nchs/nhanes/index.htm; https://www.gov.scot/collections/scottish-health-survey/. The methods and design of these surveys, as well as the characteristics of the participants, are described below.

*NHANES*: This is a national survey designed to assess the health and nutritional status of the noninstitutionalized US population using a stratified, multistage, probability sampling design, collecting data in 2‐year cycles since 1999/2000 (Kang et al. 2019). NHANES includes extensive anthropometric, socioeconomic, health, and dental examinations, as well as laboratory testing for biomarkers. Height, weight, and WC were measured onsite by trained examiners, whereas dental‐related measures were taken by a trained dental survey staff and quality controlled by a second “gold standard” examiner. NHANES 1999–2012 was used, as surveys 2013–2014 did not include dental outcomes, and we aim to match the timescale of SHeS 2014. The inclusion criteria for participants are as follows: those who are aged 19–74 years, who are not obese (BMI between 18.5 and 29.9 kg/m^2^), and with validated tooth loss records. The exclusion criteria are as follows: those under 18 years, whose BMI is either less than 18.5 or more than 29.9 kg/m^2^, or those with missing/no tooth loss records.

*SHeS*: This is a national survey that aims to provide representative data on the health of people living in Scotland. The survey uses a stratified, multistage, probability sampling design and is conducted every year, starting from 2008/2009 (Brown et al. 2015). The survey includes anthropometric, socioeconomic, health, and dental‐related examinations and questionnaires, as well as laboratory tests for participants who consent to blood examination. SHeS 2008–2014 was used, including participants satisfying the same criteria as NHANES.

### 2.2 Exposure and Outcome Variables

To make the results from NHANES and SHeS comparable, variables that were recorded differently were re‐coded in the same format to ensure consistency of measurement between the two datasets. Diet measurements in NHANES and SHeS were available but were recorded in different ways: NHANES recorded the daily sugar consumption amount using two 24‐h dietary recall interviews, whereas SHeS recorded sweets/chocolate/ice cream/soft drinks/biscuits/cakes daily eating frequency using self‐reported questionnaires. Therefore, diet was not included in this analysis due to its inconsistent measurement between the two datasets.

*Exposure*: In both NHANES and SHeS, abdominal obesity was characterized by WC. In NHANES, WC was measured by a trained examiner or nurse with a measuring tape positioned at the high point of the iliac crest. In SHeS, WC was measured by nurses palpating through clothing and identifying the point midway between the hip bone and the bottom of the lowest rib in 2008–2011; from 2012 to 2014, a trained interviewer instructed the participant to point to their navel and place the tape measure around their waist themselves. WC was classified into three levels (normal, high, and very high) for men (< 94, 94–102, and > 102 cm) and women (< 80, 80–88, and > 88 cm) according to the WHO cutoff recommendations (WC and WHR: report of a WHO expert consultation, Geneva, December 8–11, 2008, 2011). Abdominal obesity is defined as those men or women with high or very high WC. The main exposure in the study was a combination of three‐level categorized WC and two‐level BMI (18.5 ≤ BMI ≤ 24.9 kg/m^2^ and 25 ≤ BMI ≤ 29.9 kg/m^2^) among people without obesity. Due to the difference in adiposity distribution in males and females, we analyze men and women separately.

*Outcome*: Tooth loss was the main outcome. In SHeS, participants reported the number of teeth falling into one of the three categories (20 and over, 1–19, or 0 [edentulous]) through self‐completed questionnaires. In NHANES, the number of teeth was examined and reported by trained and calibrated health technologists as a count between 0 (edentulous) and 32 (full dentition). The individuals with tooth loss due to traumatic injuries were excluded. The number of remaining teeth in NHANES was further categorized the same way as the three levels in SHeS to ensure consistency and enable comparison of results. Because we have reported the effect of abdominal obesity on tooth loss using NHANES elsewhere when the number of tooth loss is treated as a continuous variable (Kang et al. 2019), in this study, we focused on the gradient effect of tooth loss severity as WCs increase.

### 2.3 Covariates

In both NHANES and SHeS, demographic, socioeconomic status (SES), lifestyle, and medical history variables for each individual were taken through onsite interviews and questionnaires. Age (years) was used as a continuous variable. The educational level was dichotomized into those with a college degree or above and into those having a high school degree or lower. Participants reported themselves as married or as single/separated. Classification of ethnicity was dichotomized as White or other race in SHeS and as non‐Hispanic White or other race in NHANES for consistency. Household income was divided into five equivalence quintiles in NHANES and SHeS, respectively. BMI (kg/m^2^) was used as a continuous variable (rounded to 0.1 kg/m^2^) and was also dichotomized into normal weight (18.5 ≤ BMI ≤ 24.9 kg/m^2^) and overweight (25 ≤ BMI ≤ 29.9 kg/m^2^).

Lifestyle variables such as alcohol intake, smoking status, and physical activity were collected through questionnaires. Participants were defined as “persons who use alcohol” if they had at least 12 alcohol drinks in the past year. Smoking status was dichotomized as “persons who ever smoked” and “persons who never smoked.” Participants were assessed as physically active (yes or no) if they had performed moderate‐intensity sports, fitness, or recreational activities that caused a small increase in breathing or heart rate for at least 10 min continuously each day. Participants reported the timing of their last dental visit (within 1 year or over 1 year) and the condition of oral hygiene (good/fair or poor). Medical conditions such as cardiovascular disease and diabetes were recorded only if they were confirmed by a doctor. CRP was determined in serum by particle‐enhanced immunonephelometry (hsCRP kit, Dade Behring) with a test sensitivity of 0.1 mg/L. CRP was categorized into three levels (< 1.0, 1.0–3.0, and > 3.0 mg/L).

### 2.4 Statistical Analysis

Descriptive statistics were reported for demographic, socioeconomic, lifestyle, anthropometric, medical conditions, and laboratory variables stratified by normal weight and overweight according to BMI and gender. Continuous variables were presented as mean (SD) or median (interquartile range), and categorical variables were reported as frequency (%).

The outcome variable for tooth loss was a categorical three‐level ordinal variable based on the number of remaining teeth (20 and over, 1–19, and edentulous). Therefore, an ordinal logistic regression was used to model the association between the severity of tooth loss and abdominal obesity measured by the categorized three‐level WC (normal, high, and very high). First, a univariable ordinal logistic regression was carried out using tooth loss as the dependent variable and a combination of BMI and WC as the independent variable. Then a multivariable ordinal logistics regression was performed using tooth loss as the dependent variable and a combination of BMI and WC as the independent variable, including demographic (age, educational level, ethnicity, and marital status), SES, lifestyle (alcohol intake, smoking status, dental visit, oral hygiene, and physical activity), medical conditions (cardiovascular diseases and diabetes), and CRP as covariates. The models were applied to men and women separately for each survey data.

As the proportion of men with normal weight and very high WC is relatively small for both datasets, sensitivity analysis by combining a high to very high WC into one category was performed. The same ordinal logistic model to the new two‐level WC variable was applied, and the results are presented in Supporting Information S1: File S1.

Both NHANES and SHeS were collected through complex survey designs that were meant to be representative of the general population in the United States and Scotland, respectively. Therefore, analysis of survey data was applied to adjust for sampling weights and incorporating clustering and stratification factors to obtain unbiased estimates and robust variance. All variables in NHANES and SHeS were re‐coded into the same format for direct comparisons but analyzed separately. Multiple imputations were conducted for missing data, and coefficients were combined using Rubin's rule. STATA 14 (StataCorp, College Station, TX, USA) and R (version 3.5.1) were used for data management and analyses. The statistical significance level was set at 0.05.

## 3 Results

NHANES consisted of 33,107 adults aged 19–74 years with BMI and WC measurements, among which 19,436 (58.7%, 9987 men and 9449 women) individuals were not obese (BMI between 18.5 and 29.9 kg/m^2^). Among people without obesity, 8874 (45.7%) were within the normal BMI range (between 18.5 and 24.9 kg/m^2^). Compared to normal‐weight individuals, overweight individuals were slightly older (mean age: 46.5 vs. 42.0 years, had a lower education level [47.4% vs. 53.4%], had a higher inflammatory CRP level [median: 1.8 vs. 1.0 mg/L], and had a higher proportion of diabetes [8.4% vs. 4.7%] and cardiovascular diseases (6.6% vs. 4.8%)] (Table 1).

**Table 1:** Characteristics of the study population from NHANES, stratified by BMI and gender.

	18.5 ≤ BMI ≤ 24.9		25 ≤ BMI ≤ 29.9	
Characteristic	Women	Men	Women	Men
*n*	4740	4134	4709	5853
Age in years, mean (SD)	41.8 (15.7)	42.3 (16.3)	45.7 (15.6)	47.1 (15.1)
Ethnicity, race other than white	2257 (47.6)	2330 (56.4)	2764 (58.7)	3233 (55.2)
Education, college or above	2775 (58.7)	1957 (47.4)	2251 (47.8)	2751 (47.0)
Single/separated	2169 (46.4)	2148 (52.6)	2124 (45.7)	2229 (38.7)
BMI, mean (SD)	22.3 (1.7)	22.7 (1.7)	27.4 (1.4)	27.5 (1.4)
Waist circumference				
Normal	2138 (46.4)	3587 (89.0)	111 (2.4)	1561 (27.4)
High	1787 (38.8)	408 (10.1)	913 (19.9)	2516 (44.2)
Very high	683 (14.8)	35 (0.9)	3558 (77.7)	1610 (28.3)
C‐reactive protein (mg/L), median (IQR)	1.1 [0.5, 2.9]	0.9 [0.4, 2.1]	2.5 [1.1, 5.1]	1.5 [0.7, 3.1]
CRP category				
< 1.0 mg/L	1730 (45.2)	1685 (52.5)	798 (20.8)	1669 (35.0)
1.0‐3.0 mg/L	1179 (30.8)	955 (29.8)	1412 (36.9)	1896 (39.8)
> 3.0 mg/L	916 (23.9)	568 (17.7)	1620 (42.3)	1203 (25.2)
Equivalized household income quintiles				
Top quintile	1334 (31.2)	870 (23.4)	938 (22.2)	1450 (27.5)
2nd quintile	897 (21.0)	744 (20.0)	952 (22.5)	1178 (22.3)
3rd quintile	908 (21.3)	896 (24.1)	978 (23.1)	1244 (23.6)
4th quintile	580 (13.6)	608 (16.3)	701 (16.6)	767 (14.5)
Bottom quintile	552 (12.9)	602 (16.2)	657 (15.5)	637 (12.1)
Last dental visits > 1 year	948 (36.1)	1281 (54.1)	1011 (39.6)	1461 (46.4)
Poor dental hygiene	405 (10.5)	506 (15.0)	474 (12.0)	593 (12.1)
Alcohol intake	2807 (66.5)	3176 (83.4)	2500 (59.0)	4647 (85.9)
Ever smoker	1794 (37.9)	2414 (58.4)	1728 (36.7)	3259 (55.7)
Physically active	2428 (51.6)	1825 (44.5)	2095 (44.9)	2644 (45.5)
Diabetes	171 (3.6)	240 (5.9)	354 (7.6)	522 (9.0)
Cardiovascular disease	165 (3.5)	261 (6.3)	246 (5.2)	453 (7.7)
Number of remaining teeth				
20 and over	4161 (87.8)	3416 (82.6)	3906 (82.9)	4849 (82.8)
19 or less	387 (8.2)	479 (11.6)	543 (11.5)	672 (11.5)
Edentulous	192 (4.1)	239 (5.8)	260 (5.5)	332 (5.7)

*Note:* Data are presented as frequency (%) unless specified. Numbers may not sum to totals due to missing values; percentages may not sum to 100 due to rounding. This table is adapted from Kang et al. (2019).

Abbreviations: BMI, body mass index; IQR, interquartile range; NHANES, National Health and Nutrition Examination Survey; SD, standard deviation.

SHeS consisted of 6107 adults between 19 and 74 years who had BMI and WC measurements, among which 4243 (69.5%, 1905 men and 2338 women) individuals were not obese (BMI between 18.5 and 29.9 kg/m^2^). Among people without obesity, 1873 (44.1%) were normal‐weight individuals. Individuals who were overweight were older (mean age: 49.8 vs. 44.5 years), had a lower level of education (31.6% vs. 37.4%), and had a higher proportion of diabetes (4.3% vs. 1.9%) and cardiovascular disease (6.7% vs. 3.7%) (Table 2).

**Table 2:** Characteristics of the study population from SHeS, stratified by BMI and gender.

	18.5 ≤ BMI ≤ 24.9		25 ≤ BMI ≤ 29.9	
Characteristic	Women	Men	Women	Men
*n*	1167	706	1171	1199
Age in years, mean (SD)	44.8 (14.5)	44.1 (15.7)	49.8 (14.2)	49.8 (14.4)
Ethnicity, race other than white	15 (2.4)	11 (3.0)	15 (2.3)	14 (2.1)
Education, college or above	451 (38.6)	250 (35.4)	371 (31.7)	378 (31.5)
Single/separated	395 (33.8)	297 (42.1)	368 (31.4)	288 (24.0)
BMI, mean (SD)	22.5 (1.6)	23.0 (1.5)	27.3 (1.4)	27.5 (1.4)
Waist circumference				
Normal	722 (61.9)	616 (87.3)	125 (10.7)	370 (30.9)
High	370 (31.7)	85 (12.0)	426 (36.4)	543 (45.3)
Very high	75 (6.4)	5 (0.7)	620 (52.9)	286 (23.9)
C‐reactive protein (mg/L), median (IQR)	0.8 [0.4, 1.7]	0.8 [0.4, 2.0]	1.4 [0.8, 2.9]	1.2 [0.6, 2.5]
CRP category				
< 1.0 mg/L	260 (55.1)	157 (54.9)	165 (32.9)	227 (42.5)
1.0‐3.0 mg/L	155 (32.8)	84 (29.4)	239 (47.6)	227 (42.5)
> 3.0 mg/L	57 (12.1)	45 (15.7)	98 (19.5)	80 (15.0)
Equivalized household income quintiles				
Top quintile	316 (29.9)	170 (26.5)	250 (23.9)	317 (29.2)
2nd quintile	224 (21.2)	140 (21.8)	245 (23.4)	266 (24.5)
3rd quintile	191 (18.1)	139 (21.7)	225 (21.5)	200 (18.4)
4th quintile	160 (15.1)	72 (11.2)	180 (17.2)	170 (15.7)
Bottom quintile	166 (15.7)	120 (18.7)	147 (14.0)	132 (12.2)
Poor oral hygiene	229 (20.8)	180 (27.3)	241 (22.7)	210 (18.7)
Alcohol intake	639 (61.8)	470 (73.2)	646 (62.5)	806 (73.8)
Ever smoker	528 (45.2)	381 (54.0)	554 (47.3)	576 (48.0)
Physically active	455 (61.6)	305 (65.6)	399 (59.1)	521 (64.5)
Diabetes	13 (1.1)	23 (3.3)	40 (3.4)	63 (5.3)
Cardiovascular disease	27 (2.3)	43 (6.1)	56 (4.8)	103 (8.6)
Number of remaining teeth				
20 and over	950 (81.4)	538 (76.2)	845 (72.2)	883 (73.6)
19 or less	122 (10.5)	80 (11.3)	171 (14.6)	162 (13.5)
Edentulous	95 (8.1)	88 (12.5)	155 (13.2)	154 (12.8)

*Note:* Data are presented as frequency (%) unless specified. Dental visit was not available. Numbers may not sum to totals due to missing values; percentages may not sum to 100 due to rounding.

Abbreviations: BMI, body mass index; IQR, interquartile range; SD, standard deviation; SHeS, Scottish Health Survey.

Compared to the NHANES cohort, the SHeS cohort was older (mean age: 47.5 vs. 44.5 years), had a lower proportion of college education level (34.2% vs. 50.1%) and abdominal obesity (23.2% vs. 31.1%), had a higher proportion of physical activity (62.5% vs. 46.7%), had a lower level of inflammatory CRP (median CRP: 1.1 vs. 1.4 mg/L), and a relatively lower prevalence of diabetes and cardiovascular disease.

**Figure 1:**
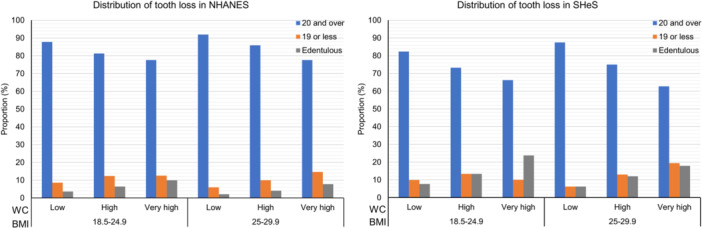
The distribution of tooth loss (number of remaining teeth 20 and over, 19 or less, and edentulous) in NHANES and SHeS populations, stratified by BMI and WC.

The distribution of tooth loss stratified by BMI and WC is shown in Figure 1 (the distributions further stratified by gender are presented in Supporting Information S2: Figures S1 and S2). There were clear trends that, in both surveys, regardless of participants being normal weight or overweight, being edentulous is positively associated with higher WCs.

The results of ordinal logistic regression in Table 3 demonstrated that compared to normal‐weight women with normal WC, normal‐weight women with high WC had a higher chance of tooth loss by 51% (odds ratio [OR]: 1.51, 95% CI: 1.14–1.99) for the NHANES cohort and by 80% (OR: 1.80, 95% CI: 1.17–2.78) for the SHeS cohort. The prevalence of tooth loss for normal‐weight women with abdominal obesity (very high WC) increased further by 64% (OR: 1.64, 95% CI: 1.16–2.34) for NHANES and 196% (OR: 2.96, 95% CI: 1.47–5.97) for SHeS, respectively. However, among women with overweight, abdominal obesity was not found to be associated with more tooth loss in both surveys.

**Table 3:** Adjusted odds ratios (95% CI) of tooth loss for women, stratified by normal/overweight population and health surveys, for predictors waist circumference, CRP, and BMI.

	18.5 ≤ BMI ≤ 24.9, OR (95% CI)		25 ≤ BMI ≤ 29.9, OR (95% CI)	
Independent variables	NHANES *n* = 4740	SHeS *n* = 1167	NHANES *n* = 4709	SHeS *n* = 1171
Waist circumference				
Normal	1	1	1	1
High	1.51 (1.14, 1.99)**	1.80 (1.17, 2.78)**	0.76 (0.35, 1.65)	0.79 (0.41, 1.50)
Very high	1.64 (1.16, 2.34)**	2.96 (1.47, 5.97)**	1.13 (0.54, 2.38)	1.12 (0.59, 2.12)
C‐reactive protein (CRP)				
< 1.0 mg/L	1	1	1	1
1.0‐3.0 mg/L	1.20 (0.91, 1.59)	1.15 (0.63, 2.11)	0.87 (0.66, 1.15)	1.40 (0.79, 2.48)
> 3.0 mg/L	1.43 (0.98, 2.10)	2.12 (0.96, 4.68)	1.07 (0.82, 1.40)	1.14 (0.58, 2.26)
BMI (kg/m^2^)	0.90 (0.83, 0.96)**	0.77 (0.68, 0.88)**	0.99 (0.93, 1.06)	1.09 (0.97, 1.23)

*Note:* Each ordinal logistic model included waist circumference as the independent variable and further adjusted for age, gender, ethnicity, education, marriage status, equivalized household income, BMI (continuous), C‐reactive protein (three levels), alcohol intake, smoking status, dental visit, oral hygiene, physical activity, diabetes, and cardiovascular disease.

Abbreviations: BMI, body mass index; CI, confidence interval; OR, odds ratio.

**p < 0.01.

The prevalence of tooth loss for normal‐weight men was not found to be associated with WC, mainly due to the small sample size in normal‐weight participants with very high WC (Table 4). However, men who were overweight with abdominal obesity had a higher prevalence of tooth loss by 41% (OR: 1.41, 95% CI: 1.06–1.87) for NHANES and 65% (OR: 1.65, 95% CI: 1.02–2.73) for SHeS compared to men who were overweight with normal WC.

**Table 4:** Adjusted odds ratios (95% CI) of tooth loss for men, stratified by normal/overweight population and health surveys, for predictors waist circumference, CRP, and BMI.

	18.5 ≤ BMI ≤ 24.9, OR (95% CI)		25 ≤ BMI ≤ 29.9, OR (95% CI)	
Independent variables	NHANES *n* = 4134	SHeS *n* = 706	NHANES *n* = 5823	SHeS *n* = 1199
Waist circumference				
Normal	1	1	1	1
High	1.62 (1.21, 2.15)**	1.26 (0.67, 2.37)	1.14 (0.89, 1.47)	1.71 (1.11, 2.63)*
Very high	1.14 (0.52, 2.49)	0.64 (0.09, 4.88)	1.41 (1.06, 1.87)*	1.65 (1.02, 2.73)*
C‐reactive protein (CRP)				
< 1.0 mg/L	1	1	1	1
1.0‐3.0 mg/L	1.53 (1.18, 2.00)**	1.08 (0.44, 2.63)	1.31 (1.04, 1.64)*	1.31 (0.77, 2.21)
> 3.0 mg/L	2.07 (1.56, 2.74)**	3.88 (1.78, 8.45)**	1.81 (1.44, 2.29)**	2.42 (1.26, 4.63)**
BMI (kg/m^2^)	0.82 (0.77, 0.87)**	0.84 (0.72, 0.97)*	0.94 (0.88, 1.01)	1.00 (0.89, 1.13)

*Note:* Each ordinal logistic model included waist circumference as the independent variable and further adjusted for age, gender, ethnicity, education, marriage status, equivalized household income, BMI (continuous), C‐reactive protein (three levels), alcohol intake, smoking status, dental visit, oral hygiene, physical activity, diabetes, and cardiovascular disease.

Abbreviations: CI, confidence interval; OR, odds ratio.

* *p* < 0.05

** *p* < 0.01.

For men who were not obese but had elevated CRP levels (> 3.0 mg/L), the chance of tooth loss nearly doubled compared to those with low CRP levels (< 1.0 mg/L) (Table 4). On the contrary, women with elevated CRP levels did not show an association with tooth loss (Table 3). Continuous BMI within each subgroup was negatively associated with tooth loss for both normal‐weight men and women, but such negative association diminished for individuals with overweight (Tables 3 and 4).

Sensitivity analysis showed that combining high and very high WC into one category did not change the direction and magnitude of the association (Supporting Information S2: Tables S1 and S2).

## 4 Discussion

Our study has revealed that higher WC in individuals without obesity is associated with an increased risk of tooth loss, and the effect is different between men and women. Specifically, abdominal obesity was associated with a higher risk of tooth loss in men who were overweight, but not in men with normal weight. Conversely, abdominal obesity was associated with a higher risk of tooth loss in women with normal weight, but not in women who were overweight.

Our research has expanded on previous studies by highlighting the importance of considering both general obesity and abdominal obesity when predicting the risk of tooth loss (Kang et al. 2019; Meisel et al. 2014; Jiang et al. 2013; Meisel et al. 2012). Previous studies have shown a weak association between tooth loss and BMI alone, with inadequate adjustments for confounding factors (Jiang et al. 2013). However, our study supports these findings by demonstrating that abdominal obesity, as measured by WC, is a significant predictor of tooth loss in both individuals with normal weight or overweight. A recent Mendelian randomization study also found a causal association between BMI and the risk of periodontal disease, but not when adjusted for WC (Dong et al. 2022). This further emphasizes the importance of considering both measures when assessing the likelihood of tooth loss.

Our study also found that the association between abdominal obesity and tooth loss is different for men and women, which is consistent with previous research (Linden et al. 2008; Meisel et al. 2014, 2019). The difference between men and women in behavior characteristics (e.g., diet and physical activity), sexual hormones, fat distribution, and inflammatory biomarkers such as CRP may cause the difference in this association. Research has shown that women tend to have higher concentrations of CRP due to greater accumulation of subcutaneous fat compared to men (Meisel et al. 2014). Abdominal obesity is closely linked to systemic inflammation as indicated by CRP levels (Cartier et al. 2009); therefore, measures such as WC in combination with BMI may provide additional information in predicting tooth loss.

It is noted that even though both NHANES and SHeS showed similar results, the population characteristics and the associated OR of tooth loss were different between the Scottish and the US populations. Scottish population have a higher prevalence of edentulous than US population (i.e., approximately 12% vs. 5%). Scotland has a history of high edentulousness dated back to the last century, and the reason behind this is a complex social (e.g., alcohol abuse) and environmental phenomenon (Clarkson and O'Mullane 1983). The prevalence of Scottish women with high or very high WC is much lower than US women in either normal or overweight groups. Therefore, a combination of factors contributed to the higher prevalence of tooth loss in Scottish women with abdominal obesity than their counterparts from the United States.

Our research has important implications for clinical practice. Despite not being considered overweight, individuals with abdominal obesity (excess abdominal fat) may still be at risk for health issues, especially for women. Current guidelines, such as the 2013 AHA/ACC Obesity Management Guidelines (Jensen et al. 2014), do not prioritize prevention programs for this population. It is important for healthcare professionals to recognize this risk and incorporate measures such as measuring WC for all patients, not just those with elevated BMI. Additionally, promoting good oral hygiene as part of a healthy lifestyle should be emphasized in response to the growing global obesity epidemic. Physical exercise is another interventional approach that reduces the risk of tooth loss as well as improves general health (Cao et al. 2023; Ferreira et al. 2019). Future studies can focus on the beneficial impact of physical activities on oral health and its associated reduction in the risk of abdominal obesity.

Our study has several strengths that enhance its validity. First, we used two large, national cohorts of participants from representative US and Scottish populations. Analysis of these separate populations yielded consistent and comparable results, bolstering the external validity of our findings. Second, WC is a reliable measure of visceral obesity and was measured simultaneously with BMI in both populations (Lean, Han, and Morrison 1995). Third, the use of inflammatory biomarkers and lifestyle factors helps to reduce the potential for confounding bias. Despite these strengths, there are also limitations to our study. As a cross‐sectional study, it is not able to prove a causal relationship between abdominal obesity and tooth loss. Additionally, self‐reported data on comorbidities, dental visits, lifestyle factors, and oral hygiene may lead to errors. Furthermore, tooth loss was self‐reported in the SHeS and classified into three levels: more than 20 teeth, 1–19 teeth, and no teeth remaining (edentulous). As a result, the quality of dental data in SHeS is not as high as in NHANES. SHeS also did not contain the same dietary record as NHANES, reporting sugar consumption frequency while NHANES recorded sugar consumption amount—this made comparing diets between Scottish and US populations impossible. Despite these limitations, the results from SHeS were consistent with those from the NHANES, in which tooth loss was recorded by trained examiners in the United States. Finally, measurement errors in our exposure variables, such as WC and BMI, may also impact our results.

## 5 Conclusions

This study has demonstrated that individuals who are not obese but have abdominal obesity face a substantially increased risk of tooth loss in both the US and Scottish populations. Additionally, the association between abdominal obesity and tooth loss was found to be distinct for men and women. These findings indicate that the impact of abdominal obesity on tooth loss in the population without obesity is significant and suggest that those individuals with abdominal obesity may be an important population for oral health prevention strategies and guideline development for public health dental care service.

## Author Contributions

J.K. contributed to the conception and design of the study, data acquisition, analysis, interpretation of data, drafting the work, reviewing it critically for important intellectual content, and final approval of the version to be published. H.L. contributed to data analysis, interpretation of data, drafting the work, and reviewing it critically for important intellectual content. S.P. contributed to the design of the work, interpretation of data, reviewing the manuscript critically, and approval of the version. J.W. contributed to the conception and design of the work, data acquisition, analysis, interpretation of data, drafting the work, reviewing it critically for important intellectual content, and final approval of the version to be published.

## Ethics Statement

This study is secondary data analysis using the National Health and Nutrition Surveys (NHANES) and the Scottish Health Survey (SHeS). Ethical approval and consent were not required as this study was based on publicly available data. Ethical approval for NHANES was conducted by the NCHS Ethics Review Board (ERB) before data collection: https://www.cdc.gov/nchs/nhanes/irba98.htm. Ethical approval for SHeS was conducted by the Health and Care Ethics Committee (reference number: 17/WA/0371) (shinyapps.io). Both ethics committees confirmed that the studies were conducted ethically in accordance with the World Medical Association Declaration of Helsinki.

## Conflicts of Interest

The authors declare no conflicts of interest.

## Supporting information

Supporting information.

Supporting information.

## Data Availability

The data that support the findings of this study are openly available in NHANES data at https://www.cdc.gov/nchs/nhanes/index.htm. Both surveys are free and publicly accessible. NHANES data can be accessed at https://www.cdc.gov/nchs/nhanes/index.htm, and SHeS data can be accessed at https://www.gov.scot/collections/scottish-health-survey/.
