# Supplementary material for: Higher Prevalence of Tooth Loss in People With Abdominal Obesity but Normal Weight: Findings From the United States and Scottish Populations
Source: Clin Exp Dent Res. 2024 Nov 19;10(6):e70047. doi: 10.1002/cre2.70047 (PMC11576517; doi:10.1002/cre2.70047)
Supplement: Supplementary file 2 — Supporting information. [file CRE2-10-e70047-s002.docx]

**Supplement materials:**

**Higher prevalence of tooth loss in people with abdominal obesity: findings from the US and Scottish non-obese population**

**Supplement Table 1**. Sensitivity analysis for adjusted odds ratios (95% CI) of tooth loss regressed on waist circumference (2-level: high/very high vs normal) for **women**, stratified by normal/overweight population and health surveys.

|  |  | 18.5≤BMI≤24.9, OR (95% CI) | |  | 25≤BMI≤29.9, OR (95% CI) | |
| --- | --- | --- | --- | --- | --- | --- |
| Independent variables |  | NHANES  n = 4740 | SHeS  n = 1167 |  | NHANES  n = 4709 | SHeS  n = 1171 |
| Waist circumference |  |  |  |  |  |  |
| Normal |  | 1 | 1 |  | 1 | 1 |
| High/Very high |  | 1.53 (1.17, 2.01)** | 1.94 (1.27, 2.95)** |  | 1.02 (0.49, 2.15) | 0.96 (0.52, 1.77) |
| C-reactive protein |  |  |  |  |  |  |
| <1.0 mg/l |  | 1 | 1 |  | 1 | 1 |
| 1.0-3.0 mg/l |  | 1.20 (0.91, 1.59) | 1.17 (0.64, 2.14) |  | 0.87 (0.66, 1.15) | 1.43 (0.81, 2.52) |
| >3.0 mg/l |  | 1.44 (1.07, 1.92)* | 2.11 (0.96, 4.63) |  | 1.09 (0.83, 1.42) | 1.22 (0.62, 2.41) |
| BMI, kg/m^2^ |  | 0.90 (0.84, 0.97)** | 0.78 (0.69, 0.88)** |  | 1.02 (0.96, 1.08) | 1.14 (1.01, 1.27)* |

OR, odds ratio; CI, confidence interval.

Each ordinal logistic model included waist circumference as independent variable, and further adjusted for age, sex, ethnicity, education, marriage status, equivalised household income, BMI (continuous), C-reactive protein (3 levels), alcohol intake, smoking status, dental visit, oral hygiene, physical activity, diabetes, cardiovascular disease.

*p<0.05; **p<0.01

**Supplement Table 2**. Sensitivity analysis for adjusted odds ratios (95% CI) of tooth loss regressed on waist circumference for (2-level: high/very high vs normal) **men**, stratified by normal/overweight population and health surveys.

|  |  | 18.5≤BMI≤24.9, OR (95% CI) | |  | 25≤BMI≤29.9, OR (95% CI) | |
| --- | --- | --- | --- | --- | --- | --- |
| Independent variables |  | NHANES  n = 4134 | SHeS  n = 706 |  | NHANES  n = 5823 | SHeS  n = 1199 |
| Waist circumference |  |  |  |  |  |  |
| Normal |  | 1 | 1 |  | 1 | 1 |
| High/Very high |  | 1.57 (1.19, 2.08)** | 1.21 (0.65, 2.23) |  | 1.20 (0.94, 1.53) | 1.69 (1.11, 2.59)* |
| C-reactive protein |  |  |  |  |  |  |
| <1.0 mg/l |  | 1 | 1 |  | 1 | 1 |
| 1.0-3.0 mg/l |  | 1.53 (1.17, 1.99)** | 3.88 (1.78, 8.45)** |  | 1.31 (1.05, 1.65)* | 1.30 (0.77, 2.20) |
| >3.0 mg/l |  | 2.05 (1.55, 2.72)** | 1.09 (0.45, 2.65) |  | 1.84 (1.45, 2.32)** | 2.41 (1.26, 4.60)** |
| BMI, kg/m^2^ |  | 0.82 (0.77, 0.87)** | 0.84 (0.72, 0.97)* |  | 0.96 (0.91, 1.02) | 1.00 (0.89, 1.12) |

OR, odds ratio; CI, confidence interval.

Each ordinal logistic model included waist circumference as independent variable, and further adjusted for age, sex, ethnicity, education, marriage status, equivalised household income, BMI (continuous), C-reactive protein (3 levels), alcohol intake, smoking status, dental visit, oral hygiene, physical activity, diabetes, cardiovascular disease. *p<0.05; **p<0.

**Supplement Figure 1**. The distribution of tooth loss for **women** in NHANES (top) and SHeS (bottom), stratified by BMI and WC.

**Supplement Figure 2**. The distribution of tooth loss for **men** in NHANES (top) and SHeS (bottom), stratified by BMI and WC.
